# Fast Simulations of Highly-Connected Spiking Cortical Models Using GPUs

**DOI:** 10.3389/fncom.2021.627620

**Published:** 2021-02-17

**Authors:** Bruno Golosio, Gianmarco Tiddia, Chiara De Luca, Elena Pastorelli, Francesco Simula, Pier Stanislao Paolucci

**Affiliations:** ^1^Department of Physics, University of Cagliari, Cagliari, Italy; ^2^Istituto Nazionale di Fisica Nucleare (INFN), Sezione di Cagliari, Cagliari, Italy; ^3^Ph.D. Program in Behavioral Neuroscience, “Sapienza” University of Rome, Rome, Italy; ^4^Istituto Nazionale di Fisica Nucleare (INFN), Sezione di Roma, Rome, Italy

**Keywords:** spiking neural network simulator, cortical microcircuits, adaptive exponential integrate-and-fire neuron model, conductance-based synapses, GPU

## Abstract

Over the past decade there has been a growing interest in the development of parallel hardware systems for simulating large-scale networks of spiking neurons. Compared to other highly-parallel systems, GPU-accelerated solutions have the advantage of a relatively low cost and a great versatility, thanks also to the possibility of using the CUDA-C/C++ programming languages. NeuronGPU is a GPU library for large-scale simulations of spiking neural network models, written in the C++ and CUDA-C++ programming languages, based on a novel spike-delivery algorithm. This library includes simple LIF (leaky-integrate-and-fire) neuron models as well as several multisynapse AdEx (adaptive-exponential-integrate-and-fire) neuron models with current or conductance based synapses, different types of spike generators, tools for recording spikes, state variables and parameters, and it supports user-definable models. The numerical solution of the differential equations of the dynamics of the AdEx models is performed through a parallel implementation, written in CUDA-C++, of the fifth-order Runge-Kutta method with adaptive step-size control. In this work we evaluate the performance of this library on the simulation of a cortical microcircuit model, based on LIF neurons and current-based synapses, and on balanced networks of excitatory and inhibitory neurons, using AdEx or Izhikevich neuron models and conductance-based or current-based synapses. On these models, we will show that the proposed library achieves state-of-the-art performance in terms of simulation time per second of biological activity. In particular, using a single NVIDIA GeForce RTX 2080 Ti GPU board, the full-scale cortical-microcircuit model, which includes about 77,000 neurons and 3 · 10^8^ connections, can be simulated at a speed very close to real time, while the simulation time of a balanced network of 1,000,000 AdEx neurons with 1,000 connections per neuron was about 70 s per second of biological activity.

## 1. Introduction

The human brain is an extremely complex system, with a number of neurons in the order of 100 billions, an average number of connections per neuron in the order of 10 thousands, hundreds of different neuron types, several types of neurotransmitters and receptors. Because of this complexity, the simulation of brain activity at the level of signals produced by individual neurons is extremely demanding, even if it is limited to relatively small regions of the brain. Therefore, there is a growing interest in the development of high-performance hardware and software tools for efficient simulations of large-scale networks of spiking neuron models. Some simulators, as for instance NEST (Fardet et al., 2020), NEURON (Carnevale and Hines, 2006), and Brian (Goodman and Brette, 2008), combine flexibility and simplicity of use with the possibility to simulate a wide range of spiking neuron and synaptic models. All three of these simulators offer support for multithread parallel computation for parallelization on a single computer. NEST and NEURON also support distributed simulations on computer clusters through MPI. On the other hand, a fertile field of research in recent decades has investigated the use of highly parallel hardware systems for simulating large-scale networks of spiking neurons. Such systems include custom made neuromorphic very-large-scale-integration (VLSI) circuits (Indiveri et al., 2011), field programmable gate arrays (FPGAs) (Wang et al., 2018), and systems based on graphical processing units (GPUs) (Sanders and Kandrot, 2010; Garrido et al., 2011; Brette and Goodman, 2012; Vitay et al., 2015; Yavuz et al., 2016; Chou et al., 2018). Compared to other highly-parallel systems, the latter have the advantages of a relatively low cost, a sustained technological development driven by the consumer market and a great versatility, thanks also to the possibility of using CUDA (Compute Unified Device Architecture), a parallel computing platform and programming model that has been created by NVIDIA to allow software developers to take full advantage of the GPU capabilities (Sanders and Kandrot, 2010). General purpose computing on graphical processing units (GPGPU) is widely employed for massively parallel computing. GPGPUs can significantly reduce the processing time compared to multi-core CPU systems for tasks that require a high degree of parallelism, because a single GPU can perform thousands of core computations in parallel. However, in order to derive maximum benefit from GPGPU, the applications must be carefully designed taking into account the hardware architecture. Over the past decade, several GPU-based spiking neural network simulators have been developed (see Brette and Goodman, 2012 for a review). EDLUT (Garrido et al., 2011) is a hybrid CPU/GPU spiking neural network simulator which combines time-driven (in GPU) and event-driven (in CPU) simulation methods to achieve real-time simulation of medium-size networks, which can be exploited in real-time experiments as for instance the control of a robotic arm. ANNarchy (Vitay et al., 2015) is a simulator for distributed rate-coded or spiking neural networks, which provides a Python interface for the definition of the networks and generates optimized C++ code to actually run the simulation in parallel, using either OpenMP on CPU architectures or CUDA on GPUs. CARLsim (Chou et al., 2018) is a GPU-accelerated library for simulating large-scale spiking neural network (SNN), which includes different neuron models and provides programming interfaces in C/C++ and in Python. Recently, the GeNN simulator (Yavuz et al., 2016; Knight and Nowotny, 2018) achieved cutting edge performance in GPU-based simulation of spiking neural networks, achieving better performance than CPU-based clusters and neuromorphic systems in the simulation of the full-scale cortical microcircuit model proposed by Potjans and Diesmann (2014). In this work we present a comprehensive GPU library for fast simulation of large-scale networks of spiking neurons, called NeuronGPU, which uses a novel GPU-optimized algorithm for spike delivery. This library can be used either in Python or in C/C++. The Python interface is very similar to that of the NEST simulator and allows interactive use of the library. Having an interface similar to that of NEST is an advantage in view of a possible integration of this library with the NEST simulator, which is currently in progress (Golosio et al., 2020). In the following sections, after a general description of the library and of the spike-delivery algorithm, we will evaluate the library on three types of spiking neural network models:

- The Potjans-Diesmann cortical microcircuit model (Potjans and Diesmann, 2014), based on the leaky-integrate-and-fire (LIF) neuron model, which describes the behavior of a region of the cerebral cortex having a surface of 1 mm^2^ and includes about 77,000 neurons and 3 · 10^8^ connections;
- A balanced network of excitatory and inhibitory neurons (Brunel, 2000), based on the adaptive-exponential-integrate-and-fire (AdEx) neuron model (Brette and Gerstner, 2005), with up to 1,000,000 neurons and 10^9^ connections;
- A balanced network of excitatory and inhibitory neurons, based on the Izhikevich neuron model (Izhikevich, 2003) and STDP synapses, with up to 1,000,000 neurons and 10^8^ connections.

We will show that, although the building time is larger compared to other simulators, NeuronGPU achieves state-of-the-art performance in terms of simulation time per unit time of biological activity.

## 2. Materials and Methods

### 2.1. The NeuronGPU Library

NeuronGPU is a GPU library for simulation of large-scale networks of spiking neurons, written in the C++ and CUDA-C++ programming languages. Currently it can simulate LIF models, different multisynapse AdEx models with current or conductance based synapses as well as user definable neuron models. The LIF model subthreshold dynamics is integrated by the *exact integration* scheme described in Rotter and Diesmann (1999) on the time grid given by the simulation time resolution. On the other hand, the numerical solution of the differential equations of the AdEx dynamics is performed through a parallel implementation, written in CUDA C++, of the fifth-order Runge-Kutta method with adaptive control of the step size (Press and Teukolsky, 1992). NeuronGPU can simulate networks of any neuron and synaptic current models whose dynamics can be described by a system of ordinary differential equations (ODEs), although currently it does not provide a dedicated interface for defining new models; the definition of a new model involves changes in specific parts of the code. However, such changes do not require experience with programming languages. In the simplest approach, the user has to modify the list of state variables and parameters, their initial values, and the differential equations that describe the neuron dynamics. With this approach the number of user-defined neuron models that can be used in a simulation together with the pre-defined models is limited to two. A more advanced approach allows to use an arbitrary number of new models in the same simulation and greater flexibility in the model definition. Detailed instructions on different approaches for the implementation of new models can be found in https://github.com/golosio/NeuronGPU/wiki/How-to-implement-new-neuron-models. The computations are carried out using mainly 32-bit floating point numerical precision, with the exception of some parts of the code for which double precision calculations are more appropriate, e.g., those in which a very large number of terms can be added. Neuron parameters and connection weights and delays can be initialized either using fixed values or through arrays or probability distributions. Neuron groups can be connected either using predefined connection rules (one-to-one, all-to-all, fixed indegree, fixed outdegree, fixed total number) or by user-defined connections. In addition to the standard synapse model, nearest-neighbor spike-timing-dependent-plasticity (STDP) is also available (Morrison et al., 2008; Sboev et al., 2016). In the STDP model, the weight that characterizes the strength of a synapse changes when the presynaptic and postsynaptic neurons emit spikes that are close in time. More specifically, the weight change depends on the time difference: Δ*t* = *t*_post_ − *t*_pre_ = *t*_spike_post_ + τ_dendritic_ − (*t*_spike_pre_ + τ_axon_) where *t*_spike_pre_ is the time the presynaptic neuron emits the spike, τ_axon_ is the axonal delay, *t*_pre_ is the time the presynaptic spike reaches the synapse, *t*_spike_post_ is the time the postsynaptic neuron emits the spike, τ_dendritic_ is the dendritic backpropagation delay, i.e., the time between the emission of the postsynaptic spike and the time in which it affects the synapse, *t*_post_ is the time in which the postsynaptic spike affects the synapse. NeuronGPU uses a symmetric-nearest-neighbor spike pairing scheme (Morrison et al., 2008). A weight change can be triggered either by the postsynaptic or by the presynaptic spike buffer. The first case occurs when the time associated with a spike stored in the postsynaptic spike buffer becomes equal to the dendritic delay. In this case Δ*t* is equal to the difference between the current time and the time in which the last presynaptic spike reached the synapse. The second case occurs when the time associated with a spike stored in the presynaptic spike buffer becomes equal to the axonal delay. In this second case, Δ*t* is equal to the difference between the time in which the last postsynaptic spike reached the input synapse and the current time. In both cases, the weight change is computed using the formula (Sboev et al., 2016):

(1) $$\Delta w=\{\begin{array}{ll} -\lambda \alpha w_{\text{max}}\cdot {\left(\frac{w}{w_{\text{max}}}\right)}^{\mu_{-}}\cdot e^{\left(\frac{\Delta t}{\tau_{-}}\right)} & \text{if }\Delta t=t_{\text{post}}-t_{\text{pre}}<\text{0}\\ \lambda w_{\text{max}}\cdot {\left(1-\frac{w}{w_{\text{max}}}\right)}^{\mu_{+}}\cdot e^{\left(-\frac{\Delta t}{\tau_{+}}\right)} & \text{if }\Delta t=t_{\text{post}}-t_{\text{pre}}>\text{0} \end{array}$$

If μ_+_ = μ_−_ = 0, the rule is called additive, while if μ_+_ = μ_−_ = 1 the rule is called multiplicative, and intermediate values are also possible. Different types of spike generators and recording devices can be simulated, including Poisson generators, spike recorders, and multimeters. NeuronGPU includes an efficient implementation of GPU-MPI communication among different nodes of a GPU cluster, however the performance of the proposed library on GPU clusters has not yet been thoroughly evaluated, therefore this feature is not described in the present work. The Python interface is very similar to that of NEST in main commands, use of dictionaries, connection rules, model names, and parameters. The following Python code sample illustrates this strong similarity.


  import neurongpu as ngpu
  # create Poisson generator with rate
  poiss_rate
  pg = ngpu.Create(‘‘poisson_generator'')
  poiss_rate = 12000. 0
  ngpu. SetStatus(pg, ‘‘rate,'' poiss_rate)
  # Create n_neurons neurons with n_receptor
  receptor ports
  # neuron model is multisynapse AdEx (aeif)
  with conductance-based synapse
  # described by the beta function
  n_neurons = 10
  n_receptor = 2
  neuron = ngpu.Create(‘‘aeif_cond_beta,''
  n_neurons, n_receptors)
  # Initialize receptor parameters
  E_rev = [0.0, -85.0]
  tau_decay = [1.0, 1.0]
  tau_rise = [1.0, 1.0]
  ngpu.SetStatus(neuron,
  {‘‘E_rev'':E_rev, ‘‘tau_decay'':tau_decay,
  ‘‘tau_rise'':tau_rise})
  # Connect Poisson generator to neurons
  poiss_weight = 0.05
  poiss_delay = 2.0
  conn_dict={‘‘rule'': ‘‘all_to_all''}
  syn_dict={‘‘0weight''1: poiss_weight, ‘‘2delay''3:
  poiss_delay, ‘‘4receptor''5:0}
  ngpu.Connect(poiss_gen, neuron, conn_dict,
  syn_dict)


About 30 test scripts and C++ programs have been designed to check the correctness of neuron model dynamics, spike generators, recording tools, spike delivery, connection rules. Many of such tests use similar NEST simulations as reference. Several examples in C++ and in Python are also available. NeuronGPU is an open-source library, freely available on GitHub from the web address https://github.com/golosio/NeuronGPU under the terms of the GNU General Public License v3.0.

### 2.2. The Spike-Delivery Algorithm

A crucial issue that must be addressed in the design of spiking neural network simulators is the choice of the algorithms to store the spikes and to propagate and deliver them after proper delays. In particular, two important aspects can significantly affect the performance of different approaches: the way they account for the delays associated with connections and the representation used to index connections and to retrieve them when they must be used for spike delivery. A common approach for handling delays consists in using a circular event queue (see for instance Brette et al., 2007). Each element of this queue corresponds to a time index, and points to a list of synaptic spikes that are scheduled for that time. When a neuron *i* fires a spike, for each target neuron *j* a synaptic event *i* → *j* is scheduled to be delivered at a time *t* + *d*, where *d* is the synaptic delay. The computational cost per time step of managing delays with this approach is (Brette et al., 2007)

(2) $$\begin{array}{l} c_{d}\times N\times F\times C\times dt \end{array}$$

where *c*_*d*_ is the cost of one store and retrieve operation in the circular queue, *N* is the number of neurons or other spiking devices, *F* is the average firing rate, *C* is the number of output connections per neuron and *dt* it the simulation time step. The computational cost per time step for propagating the spikes is

(3) $$\begin{array}{l} c_{p}\times N\times F\times C\times dt \end{array}$$

where *c*_*p*_ is the cost of one spike propagation. In CPU implementations of this approach, *c*_*d*_ is usually small compared to *c*_*p*_, therefore handling delays through the circular queue increases the cost of spike propagation by a small factor. On the other hand, in a GPU implementation *c*_*d*_ may not be small compared to *c*_*p*_, because the insertion and retrieval operations in the circular queue would require access to the GPU global memory. This type of access is relatively slow, and represents in many cases one of the main bottlenecks of GPU codes. For this reason, many GPU-based simulators use different methods. Nageswaran et al. (2009) propose an approach for handling spikes and synaptic delays in GPU architectures based on two tables: a firing count table and a firing address table. The firing count table stores the cumulative count of neurons that emitted a spike in each time step of the last second. The firing address table holds the indexes of the neurons that emitted a spike in the last second. The firing count table is used to retrieve from the firing address table the list of all the neurons that fired in each time step *t*′, with *t* − max_delay ≤ *t*′ ≤ *t*, where *t* is the current time step, and max_delay is the maximum delay of all synaptic connections of the network, expressed in time step units. The computational cost per time step for retrieving the spikes emitted in that interval is *O*(*N* × *F* × max_delay × *dt*). The spikes emitted in the time step *t*′ are sent to the neurons' outgoing synaptic connections having a delay equal to *t* − *t*′. Synaptic connections are represented through a sparse representation similar to adjacency lists for directed graphs. Each neuron has a list of output connections, identified by the index of the target neuron and by the index of the synapse in that neuron. The connections in the list are sorted based on their delays. Two arrays, delay start and delay count, are used to retrieve the connections corresponding to a given delay: delay start[*k*] is the index of the first connection in the list with a delay of *k* ms, and delay count[*k*] is the number of connections having that delay. A drawback of this approach is that spikes produced by neurons that have outgoing connections with a maximum delay much less than max_delay remain in the firing address table and are retrieved for a number of time steps equal to max_delay.

Yavuz et al. (2016) propose an algorithm for handling spikes and synaptic delays based on a circular queue array structure, with *N* × *m* elements, where *m* = delay/*dt*. An index *p* points to the slots of the queue, and is increased by 1 at every time step. A spike of the *i*th neuron is stored in the slot (*i, p*) of the queue, and spikes to be delivered are retrieved from the slots [*i*, (*p* − *m*) mod *m*]. This approach is very efficient, with a computational cost *O*(*N*), however it has the limitation that delays have to be identical across the synapses of each synapse population. In order to use different delays, a synapse population has to be defined for each delay, with its own circular queue structure. In particular, this approach would not be efficient in realistic conditions where the delays vary according to some probability distribution. The spikes retrieved from the queue are delivered to the target neurons through a connection matrix, either an all-to-all connection matrix in case of dense connections, or based on the YALE sparse matrix format (Eisenstat et al., 1982) in case of sparse connectivity.

**Figure 1:**
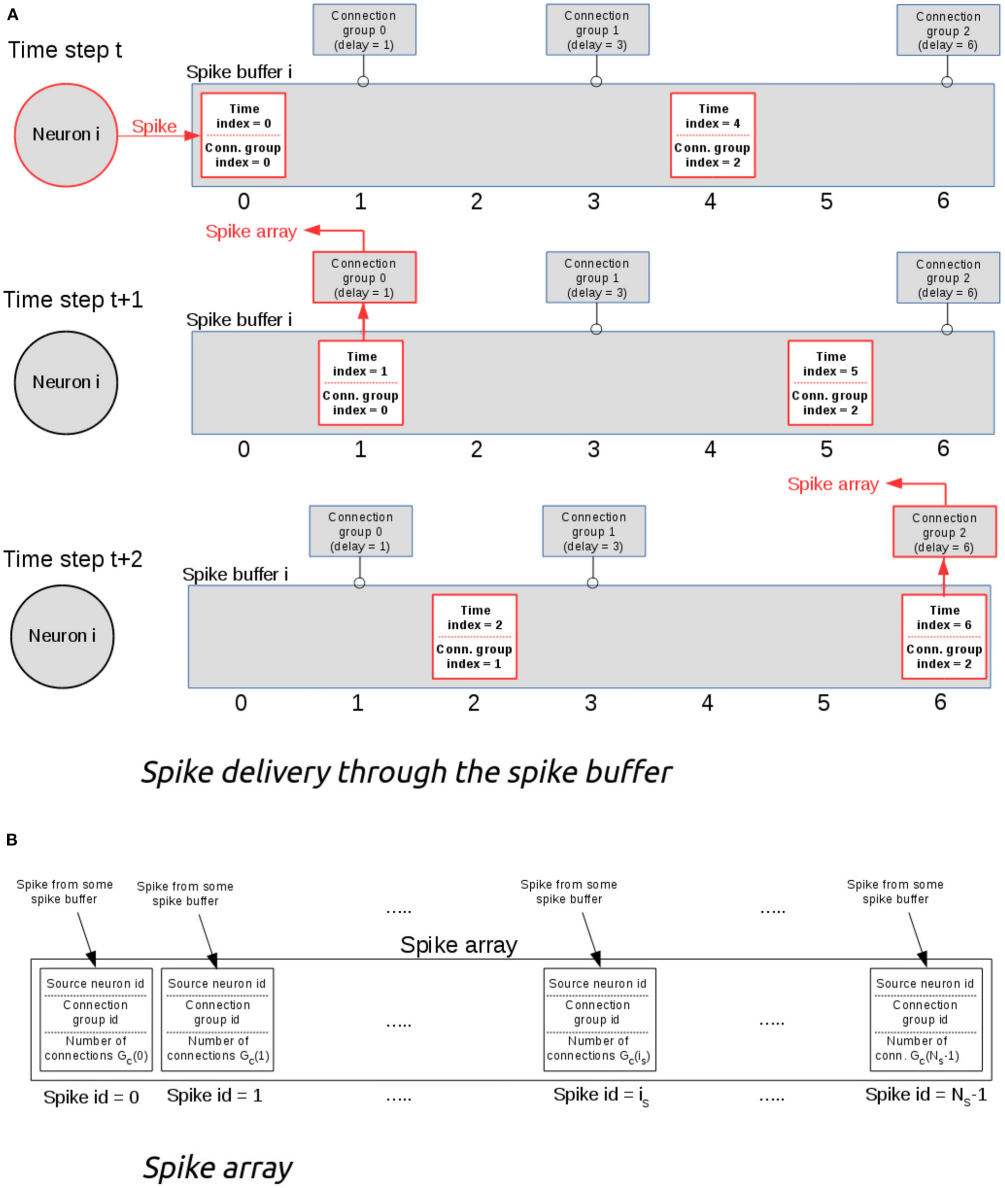
**(A)** Example of spike delivery through the spike buffer. At time t, the i-th neuron emits a spike which is inserted in the spike buffer. In this example, the buffer contains also another spike emitted previously. At each time step, the spike time index is incremented by 1. When it becomes equal to the delay of some connection group, the spike is delivered to that group and its connection group index is incremented by 1. **(B)** The spike array. When the time index of a spike matches the delay of a connection group, the spike is sent to the spike array, which is used for delivering the spike to all neurons of the connection group.

NeuronGPU uses one (output) spike buffer per neuron, which holds the spikes that have been fired by the neuron. The output connections of each neuron are organized in groups, all connections in the same group having the same delay (see Figure 1). Only three values per spike are stored in the buffer: a multiplicity, a time index *t*_*s*_, which starts from 0 and is incremented by 1 at every time step, and a connection-group index *i*_*g*_, which also starts from zero and is incremented by 1 every time the spike reaches a connection group, i.e., when the time index *t*_*s*_ matches the connection-group delay. Figure 1A represents the structure of the spike buffer and illustrates an example of how the spike is delivered from the neuron that fired it to the target neurons of different connection groups. Keeping a connection-group index and having output-connection groups ordered according to their delays is useful for reducing the computational cost, because with this approach there is no need for a nested loop for comparing the time index of the spike with the connection delays. When the time index of a spike *t*_*s*_ matches a connection-group delay, the spike is sent to the spike array, as shown in Figure 1B. Finally, spikes are sent from this array to the target neurons. This final delivery is done directly by a CUDA kernel, so no additional memory is required. The maximum size of the global spike array is equal to the number of nodes (i.e., neurons and other spiking devices), so the maximum GPU memory required by this algorithm is well-defined.

In MPI connections, when a source node (a neuron or another spiking device) is connected to target nodes of another host, a spike buffer, similar to the local one, is created in the remote host. When the source node fires a spike, this is sent to its spike buffer of the remote host, which delivers the spike to all target neurons after proper delays.

The computational cost per time step of the spike-buffer update algorithm is *c*_*s*_ × *N* × *B*, where *c*_*s*_ is the cost of a single spike update and *B* is the average number of spikes stored in a spike buffer. If we call *d*_max_(*i*) the maximum delay, expressed in time step units, of the outgoing synaptic connections of the *i*th neuron, and 〈*d*_max_(*i*)〉 its average over all the neurons, *B* can be expressed as

(4) $$\begin{array}{l} B=F\times \langle d_{\text{max}}\left(i\right)\rangle \times dt \end{array}$$

and therefore the cost of the spike buffer update is

(5) $$\begin{array}{l} c_{s}\times N\times F\times \langle d_{\text{max}}\left(i\right)\rangle \times dt \end{array}$$

It should be observed that 〈*d*_max_(*i*)〉 is less than or equal to max_delay, which is the maximum delay of all synaptic connections of the network and can be expressed as max_delay = max_*i*_{*d*_max_(*i*)}, therefore the order of the computational cost of the proposed approach is smaller than or equal to that proposed by Nageswaran et al.

The computational cost per simulation time step for writing and reading the spikes to and from the spike array is *O*(*N* × *F* × *dt*). This contribution is usually much smaller than the cost of neuron dynamics update, which is *O*(*N*), because in realistic conditions *F* × *dt* ≪ 1. The computational cost per simulation time step for delivering the spikes from the spike array to the target neurons is

(6) $$\begin{array}{l} c_{d}\times N\times F\times C\times dt \end{array}$$

where *c*_*d*_ is the cost for delivering a single spike. By comparing this cost with that of the spike buffer update, it can be observed that when

(7) $$\begin{array}{l} C\gg \langle d_{\text{max}}\left(i\right)\rangle \times c_{s}/c_{d} \end{array}$$

the delivery of the spikes to the target neurons gives the main contribution to the computational cost. This is usually the case when the number of connections per neuron is of the order of hundreds or more. An advantage of the proposed approach is that the delivery of the spikes from the spike array to the target neurons requires a small number of global memory accesses per delivery, therefore *c*_*d*_ is relatively small.

### 2.3. The Potjans-Diesmann Cortical Microcircuit Model

**Figure 2:**
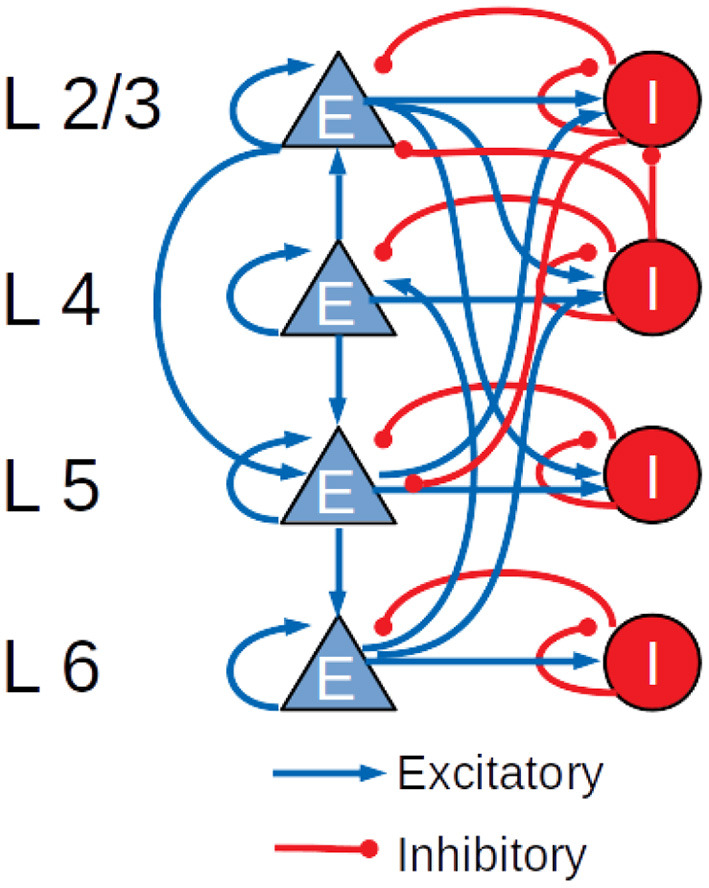
Schematic diagram of the Potjans-Diesmann cortical microcircuit model.

The cortical microcircuit model used in this work was developed in 2014 by Potjans and Diesmann (2014) and describes a portion of 1 mm^2^ of sensory cortex, comprising approximately 77,000 LIF neurons organized into layers 2/3, 4, 5, and 6. Each layer contains an excitatory and an inhibitory population of LIF neurons with current-based synapses, for a total of eight populations: 2/3I, 2/3E, 4I, 4E, 5I, 5E, 6I, and 6E. The number of neurons in each population, the connection probability matrix and the rates of the external Poisson inputs are based on the integration of anatomical and physiological data mainly from cat V1 and rat S1. The total number of connections is about 3 · 10^8^. Figure 2 shows a diagram of the model with a schematic representation of the connections having probabilities >0.04.

The LIF neuron model, used in the cortical microcircuit, is one of the simplest spiking neuron models. The neuron dynamics is modeled by the following differential equation

(8) $$\begin{array}{l} \tau_{m}\frac{dV_{i}}{dt}=-\left(V_{i}-E_{L}\right)+R_{m}I_{\text{syn},i} \end{array}$$

where *V*_*i*_(*t*) represents the membrane potential of neuron *i* at time *t*, τ_*m*_ is the membrane time constant, *E*_*L*_ is the resting membrane potential, *R*_*m*_ is the membrane resistance and *I*_syn,*i*_ is the synaptic input current. In the exponential shaped postsynaptic currents (PSCs) model, which will be used to simulate the Potjans-Diesmann cortical microcircuit model, the input current is described by the following equation

(9) $$\begin{array}{l} \tau_{\text{syn}}\frac{dI_{\text{syn},i}}{dt}=-I_{\text{syn},i}+\underset{j}{\sum }w_{ij}\underset{t_{j}^{f}}{\sum }\delta \left(t-t_{j}^{f}\right) \end{array}$$

where τ_syn_ is the synaptic time constant, *w*_*ij*_ are the connection weights and $t_{j}^{f}$ are the spike times from presynaptic neuron *j*. The simulation time step is set to 0.1 ms.

### 2.4. The AdEx-Neurons Balanced Network Model

**Figure 3:**
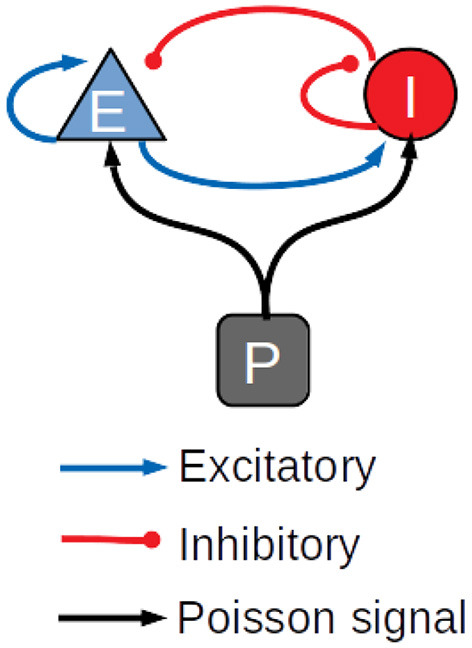
Schematic diagram of the balanced network used in the simulations.

The performance of the library was also assessed on a balanced network of sparsely connected excitatory and inhibitory neurons (Brunel, 2000), using the AdEx neuron model with conductance-based synapses and synaptic conductance modeled by an alpha function (Roth and van Rossum, 2013). The differential equations underlying the neuron dynamics are solved using the fifth-order Runge Kutta method with adaptive step size. To our knowledge, other GPU simulators of large scale spiking neural networks do not support this method. For this reason, the results of the simulations of the AdEx-neurons balanced network model are compared only with the CPU-based simulator NEST, which supports the same method. In general, GPU simulations work more efficiently with fixed step size; the adaptive step size is challenging and it was not obvious a priori that a GPU simulator could be faster than multi-core CPU systems with this kind of methods. Both populations of excitatory and inhibitory neurons are stimulated by an external Poissonian signal, as shown in Figure 3. Simulations have been made with a variable number of neurons and connections, with up to 1,000,000 neurons and 10^9^ connections. Table 1 represents the parameters used for the balanced network simulations.

**Table 1:** Values of the parameters used for the balanced network simulations.

**Parameter**	**Value**
*N*_*ex*_ (n. of excitatory neurons)	Variable
*N*_*in*_ (n. of inhibitory neurons)	*N*_*ex*_/4
*CE* (n. of input excitatory synapses per neuron)	Variable
*CI* (n. of input inhibitory synapses per neuron)	*CE*/4
*W*_*ex*_ (excitatory connection weight)	0.05
*W*_*in*_ (inhibitory connection weight)	0.35
Mean delay	0.5 ms
Delay STD	0.25 ms
*W*_*poisson*_ (Poisson signal weight)	0.37
*Rate*_*poisson*_ (Poisson signal rate)	20,000 Hz
Neuron average firing rate	30.7 Hz

The AdEx model represents an attractive neuron model for use in large-scale network simulations, because it is relatively simple compared to biologically detailed spiking neuron models, nonetheless it provides a good level of realism in representing the spiking behavior of biological neurons in many conditions, in the sense that it fits well the response of neurons as measured from electrophysiological recordings (Brette and Gerstner, 2005). This model is described by a system of two differential equations. The first equation describes the dynamics of the membrane potential V(t) and includes an activation term with an exponential voltage dependence

(10) $$\begin{array}{l} C\frac{dV}{dt}=-g_{L}\left(V-E_{L}\right)+g_{L}\Delta_{T}e^{\frac{V-V_{T}}{\Delta_{T}}}+I_{syn}\left(V,t\right)-\omega +I_{e} \end{array}$$

where the synaptic current is

(11) $$\begin{array}{l} I_{\text{syn}}\left(V,t\right)=\underset{i}{\sum }g_{i}\left(t\right)\left(V-E_{\text{rev},i}\right) \end{array}$$

*C* is the membrane capacitance, *g*_*L*_ is the leak conductance, *E*_*L*_ is the leak reversal potential, Δ_*T*_ is a slope factor, *V*_*T*_ is the spike initiation threshold, ω is the spike-adaptation current, *I*_*e*_ is an external input current, *g*_*i*_(*t*) are the synaptic conductances and *E*_rev,*i*_ are the reversal potentials. The voltage is coupled to a second equation which describes adaptation

(12) $$\begin{array}{l} \tau_{\omega }\frac{d\omega }{dt}=a\left(V-E_{L}\right)-\omega \end{array}$$

where τ_ω_ is the adaptation time-constant and *a* is the subthreshold adaptation parameter. When the neuron fires a spike, the adaptation current ω changes into ω → ω + *b*, where *b* is a spike-triggered adaptation parameter, while the membrane potential changes into *V* → *V*_*r*_. Table 2 reports the AdEx parameter values that have been used for the balanced network simulations. The time step for spike communication is set to 0.1 ms.

**Table 2:** Values of the AdEx parameters used in the balanced network simulations.

**Parameter**	**Value**
*C* (Membrane capacitance)	281 pF
*g*_*L*_ (leak conductance)	30 nS
*E*_*L*_ (leak reversal potential)	−70.6 mV
*V*_*T*_ (spike initiation threshold)	−50.4 mV
Δ_*T*_ (slope factor)	2 mV
τ_*w*_ (adaptation time constant)	144 ms
*a* (subthreshold adaptation)	4 nS
*b* (spike-triggered adaptation)	80.5 pA
*V*_*r*_ (reset value of *V*_*m*_ after a spike)	−60 mV
*E*_*ex*_ (excitatory reversal potential)	0 mV
*E*_*in*_ (inhibitory reversal potential)	−85 mV
τ_syn_ (synaptic time constant)	1 ms

### 2.5. The Izhikevich-Neurons Balanced Network With STDP Synapses

The architecture of this model is still that shown in Figure 3 and the ratio of excitatory to inhibitory neurons is the same as the model presented in the previous section. The other features of the model are listed below:

- Time step of 1 ms;
- 4-parameters Izhikevich neuron model (Izhikevich, 2003);
- Current-based synapses described by an exponential-decay function;
- Euler forward integration method with two integration steps per simulation time step;
- 100 connections per neuron;
- Excitatory synapses change their weights according to the STDP rule, while inhibitory synapses have fixed weights;
- Average firing rate of 16 Hz for both excitatory and inhibitory populations.

The values of the Izhikevich-neuron parameters are *a* = 0.02, *b* = 0.2, *c* = −65, and *d* = 8. The synaptic decay time is τ_decay_ = 2 ms. Table 3 reports the values of the STDP parameters. The value of λ is small so that the weights do not change significantly during the simulation. It should be considered that the simulation time overhead due to STDP synapses depends only on the spike time distributions and not on the values of the STDP parameters if λ is sufficiently small.

**Table 3:** Values of the STDP parameters used in the Izhikevich-neurons balanced network simulations.

**Parameter**	**Value**
τ_+_	20.0 ms
τ_−_	20.0 ms
λ	0.001
α	1.0
μ_+_	1.0
μ_−_	1.0
*w*_max_	10.0

## 3. Results

The cortical microcircuit model and the balanced network described in the previous section were used both to verify the correctness of the simulations performed using NeuronGPU and to compare the performance of the proposed library with those of NEST version 2.20.0 (Fardet et al., 2020) and GeNN version 3.2.0 (neworderofjamie et al., 2018). For this purpose, we used a PC with a CPU Intel Core i9-9900 K with a frequency of 3.6 GHz and 8 cores featuring hyperthreading with two threads per core, for a total number of 16 hardware threads, 64 GB RAM, and a GPU card NVIDIA GeForce RTX 2080 Ti with 11 GB of GDDR6 VRAM, 4,352 CUDA cores, and a boost clock of 1,635 MHz. NeuronGPU and GeNN simulations were also performed on a system equipped with an NVIDIA Tesla V100 GPU with 16 GB GPU memory and 5,120 CUDA cores.

### 3.1. Simulation of the Cortical Microcircuit Model

Following the procedure proposed by van Albada et al. (2018) and by Knight and Nowotny (2018), in this section we will verify the correctness of the simulations by comparing some relevant statistical distributions extracted from the simulations of the Potjans-Diesmann cortical microcircuit model made using NeuronGPU with the analogous distributions obtained using the NEST simulator. Subsequently, still following the same line of van Albada et al. (2018) and Knight and Nowotny (2018), the cortical microcircuit model will be used as a benchmark to evaluate the performance of NeuronGPU in terms of building time and simulation time per unit time of biological activity.

**Figure 4:**
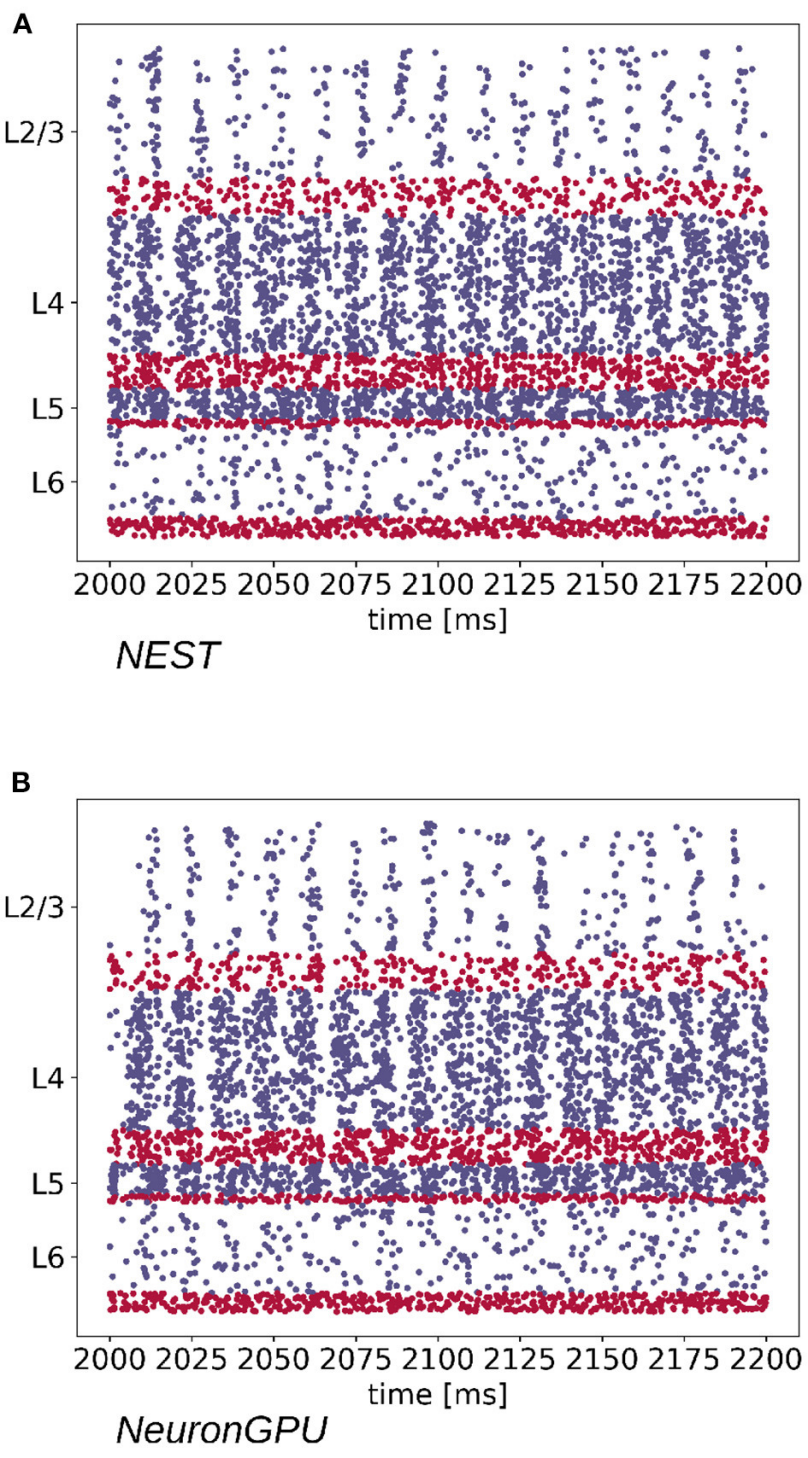
Raster plot showing spike times (dots) of neurons from each population of the cortical microcircuit model, simulated using **(A)** NEST and **(B)** NeuronGPU, in a time window of 200 ms (in blue the excitatory and in red the inhibitory). Due to the high number of neurons in the model, only the spikes of one neuron out of ten are shown.

The Python code used for simulations, available in https://github.com/golosio/NeuronGPU/tree/master/python/Potjans_2014, is almost identical to the NEST implementation (https://nest-simulator.readthedocs.io/en/stable/microcircuit/). Figure 4 shows a raster plot of the spike times of neurons from each population of the model, simulated using NEST and NeuronGPU, in a time window of 200 ms.

In order to verify the correctness of the simulations, we simulated 11 s of biological activity of the full-scale Potjans-Diesmann model with both NeuronGPU and NEST, with a time step of 0.1 ms. For both simulators we performed 10 simulations, distinct from each other only for the initial seed for random number generation. As in van Albada et al. (2018) and Knight and Nowotny (2018), the first second was discarded in order to eliminate transient trends. The spike times of all neurons have been recorded during the simulations, and subsequently they have been used to extract three distributions for each population, namely:

- The average firing rate of the single neuron;
- The coefficient of variation of the inter-spike time interval (CV ISI), defined as the ratio between the standard deviation and the average of the inter-spike time intervals;
- The Pearson correlation between the spike trains.

The latter has been computed on a subset of 200 neurons for each population, as in van Albada et al. (2018) and Knight and Nowotny (2018). This number represents a compromise between statistical precision and computation time. The spike trains of those neurons have first been rebinned to a time step of 2 ms, equal to the refractory time. Denoting the binned spike trains as *b*_*i*_ and their mean value as μ_*i*_, the correlation coefficient between the spike trains *b*_*i*_ and *b*_*j*_ is defined as

$$\begin{array}{l} C\left[i,j\right]=<b_{i}-\mu_{i},b_{j}-\mu_{j}>/\\ \;\sqrt{<b_{i}-\mu_{i},b_{i}-\mu_{i}>\cdot <b_{j}-\mu_{j},b_{j}-\mu_{j}>} \end{array}$$

where < , > represents the scalar product. For 200 spike trains, a 200x200 correlation matrix is returned. The Pearson correlation distribution is evaluated as the distribution of the off-diagonal elements of this matrix. All distributions have been evaluated from the spike time recordings using the Elephant (Electrophysiology Analysis Toolkit) package (Denker et al., 2018), dedicated to the analysis of electrophysiological data in the Python environment. The distributions have been smoothed using the KDE (Kernel Density Estimation) method (Rosenblatt, 1956; Parzen, 1962), available in the *scikit-learn* Python library (Pedregosa et al., 2011) through the function sklearn.neighbors.KernelDensity. The KDE method allows to estimate the probability density of a random variable with a reduced dependence on random fluctuations linked to individual simulations. In particular, each of the *N* points belonging to a sample is represented by a Gaussian function of suitable width, called kernel bandwidth. The integral of each of these functions is normalized to 1/*N*; the overall distribution is therefore estimated as the sum of all these Gaussians, and obviously it has an integral normalized to one. The kernel bandwidth has been optimized using the so-called Silverman's rule (Silverman, 1986), which prescribes a bandwidth value of

(13) $$\begin{array}{l} b=0.9\cdot \min\left(\hat{\sigma },\frac{\text{IQR}}{1.349}\right)\cdot N^{-\frac{1}{5}} \end{array}$$

where $\hat{\sigma }$ is the standard deviation of the samples, *N* is the sample size and IQR is the interquartile range. It should be observed that the distributions obtained through the KDE method are continuous functions, since they are evaluated as the sum of a set of Gaussian functions.

**Figure 5:**
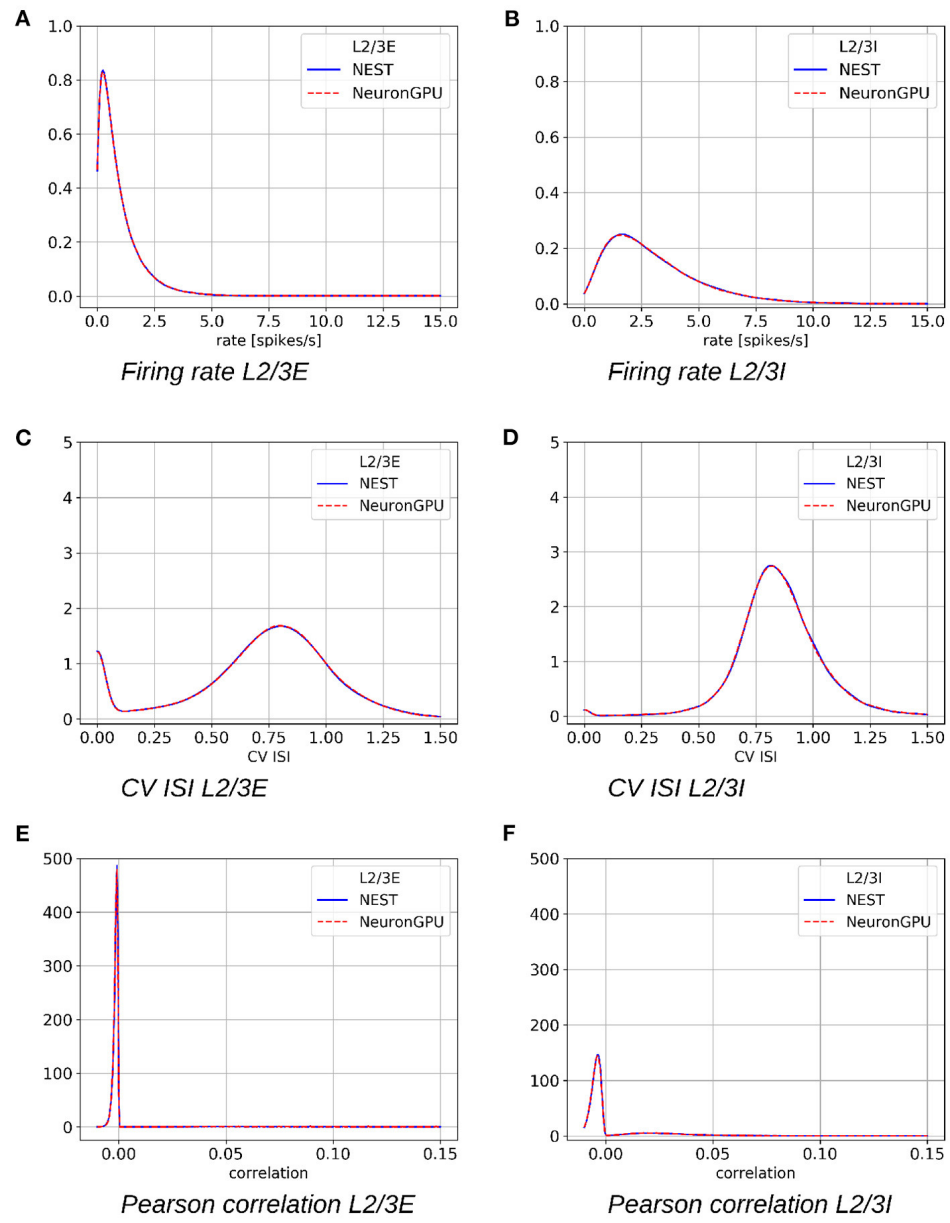
Distribution of the firing rates, coefficient of variation of interspike intervals (CV ISI) and Pearson correlation coefficient of the spike trains for the populations L2/3E and L2/3I of the cortical microcircuit model, averaged over 10 simulations, made using NEST (blue) or NeuronGPU (red). **(A)** Firing rate L2/3E, **(B)** firing rate L2/3I, **(C)** CV ISI L2/3E, **(D)** CV ISI L2/3I, **(E)** Pearson correlation L2/3E, **(F)** Pearson correlation L2/3I.

**Figure 6:**
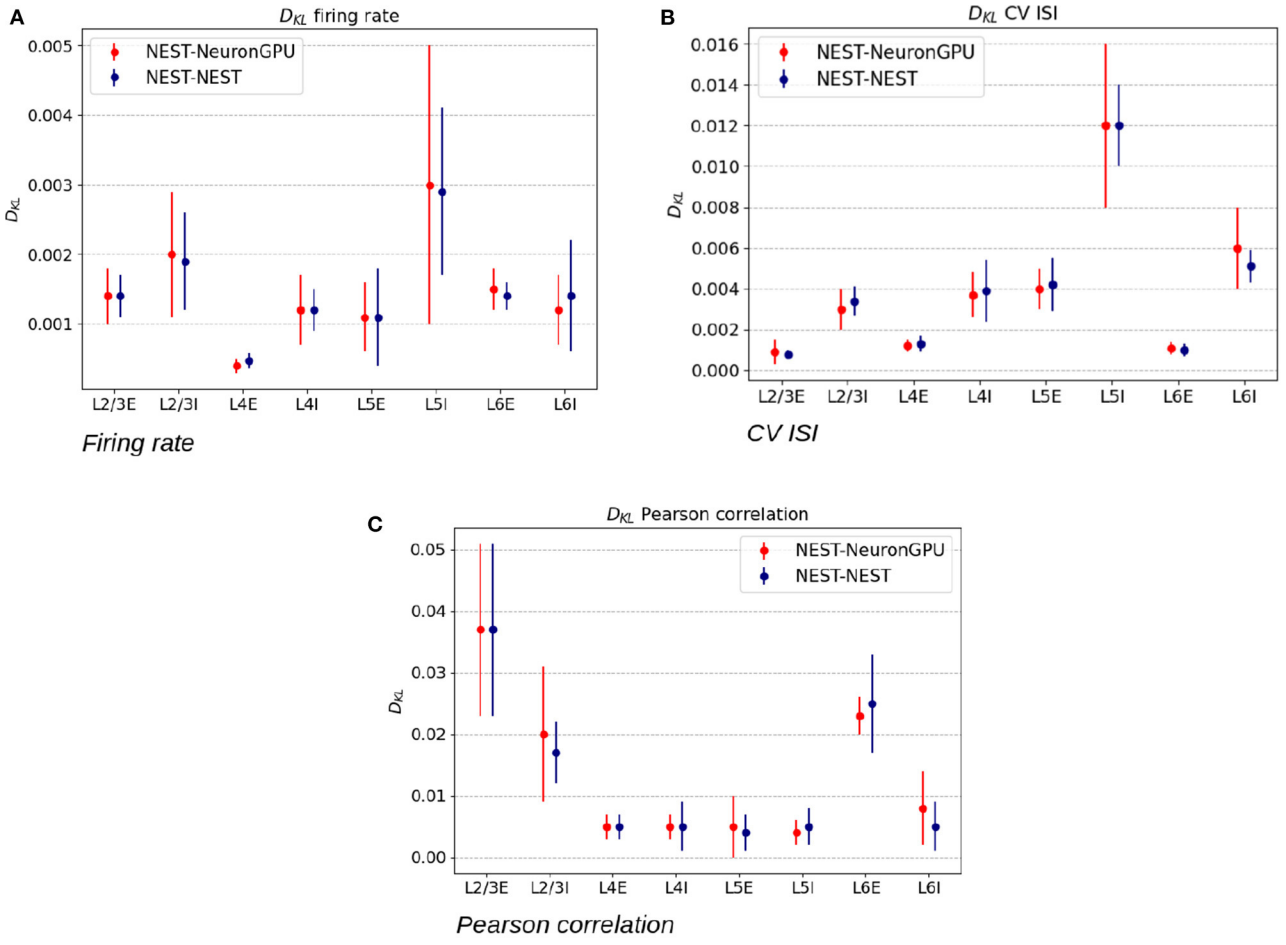
Kullback-Leibler divergence between the distributions of the firing rate **(A)**, coefficient of variation of interspike intervals **(B)**, and Pearson correlation coefficient **(C)**, extracted from NEST and NeuronGPU simulations. The red error bars represent the average values and the standard deviations of the divergence between NEST and NeuronGPU, while the blue ones represent the same values for NEST simulations with different seeds.

**Figure 7:**
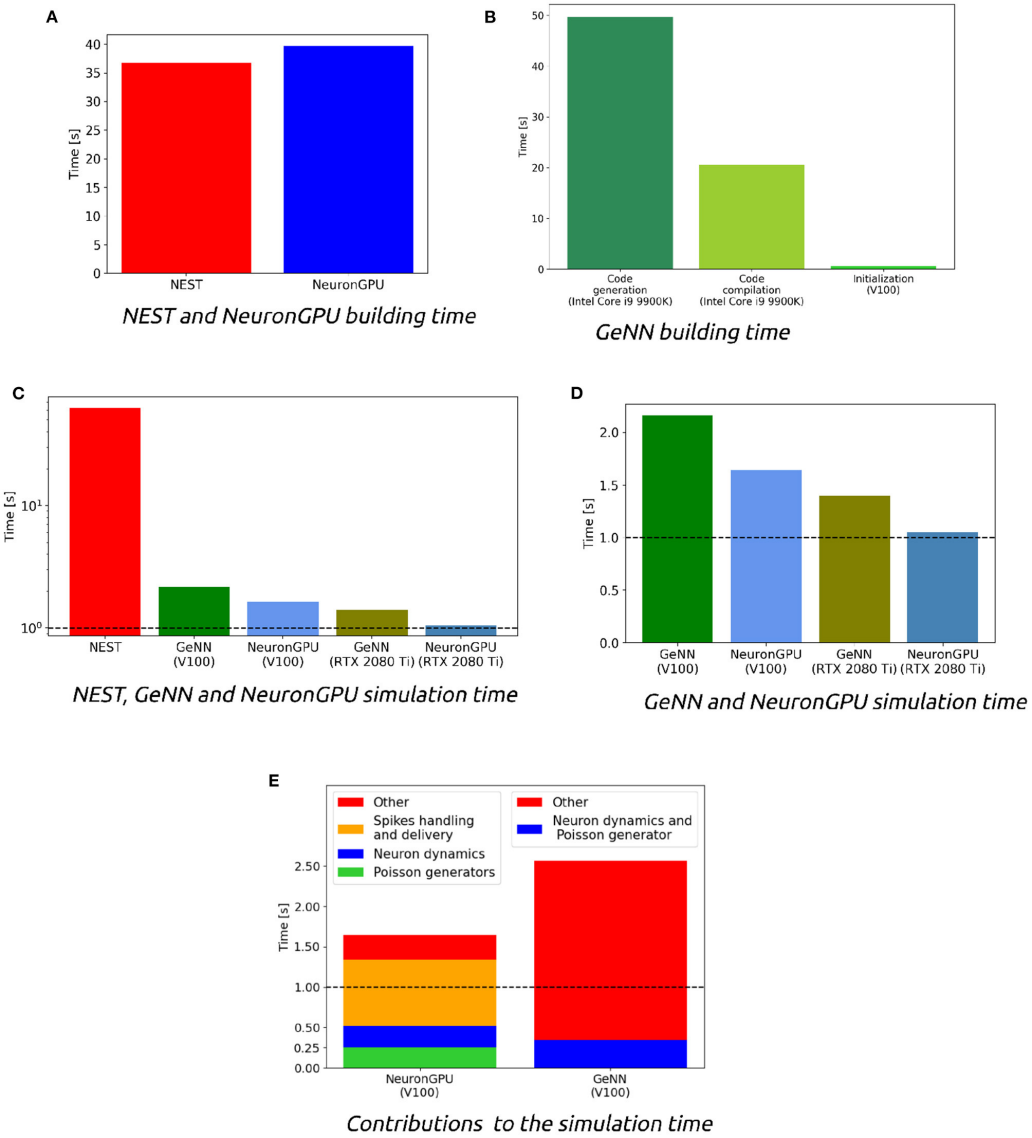
**(A)** Building time of the cortical microcircuit model simulated with NEST and with NeuronGPU on a system equipped with an Intel Core i9-9900K CPU. **(B)** Times for code generation, compilation, and initialization of the cortical microcircuit model for GeNN. The first two phases are performed by the CPU, and the times refer to a system equipped with an Intel Core i9-9900K. The third phase is mainly performed by the GPU, and the figure shows the time for an NVIDIA Tesla V100. **(C,D)** Simulation times per second of biological time of the cortical microcircuit model simulated with NEST, NeuronGPU, and GeNN on various CPU and GPU hardware. **(E)** Contributions of neuron dynamic update time, Poisson generator time, and spike handling and delivery time to the total simulation time of the Potjans-Diesmann model, simulated using NeuronGPU and GeNN on a Tesla V100 GPU. In GeNN the Poissonian input signal is generated within the same code that manages the neuron's dynamics, and furthermore it was not possible to separate the time used for spike handling and delivery from the remaining contributions to the simulation time. The horizontal line represents the biological time. The simulation time step is set to 0.1 ms.

Figure 5 shows the distributions of the firing rate, the CV ISI and the Pearson correlation coefficient for two populations of the Potjans-Diesmann model, averaged over 10 simulations made using NEST or NeuronGPU. As can be seen in the graphs, the distributions obtained from the two simulators are very similar to each other. This is also true for the other populations of the model. In order to compare quantitatively the distributions obtained using NeuronGPU to those obtained using NEST, we evaluated the Kullback-Leibler (KL) divergence (Kullback and Leibler, 1951), defined as $D_{KL}\left(p_{1},p_{2}\right)=-\underset{i}{\sum }p_{1,i}\log\left(p_{1,i}/p_{2,i}\right)$, where *p*_1_ and *p*_2_ are two distributions, and the index *i* runs on the sampling points of the two distributions. For this purpose, we used 10 pairs of simulations (NeuronGPU-NEST and NEST-NEST) using different seeds for random number generation. The KL divergence was then calculated for each pair and its average and standard deviation were calculated on the 10 pairs. Since the KDE method provides a smooth continuous function, the result is not sensitive to the sampling step as long as this is small enough. The KL divergence was evaluated using the Python scientific library (Virtanen et al., 2020) and in particular the scipy.stats.entropy function. Figure 6 shows the average and standard deviation of the KL divergences between the distributions of firing rates, CV ISI, and Pearson correlation, obtained from NEST and from NeuronGPU simulations, for the eight populations of the cortical microcircuit model. It can be observed that the KL divergence between distributions obtained from NEST and from NeuronGPU are perfectly compatible with the divergence between distributions obtained from NEST simulations with different seeds. To compare the performance of NeuronGPU with those of NEST and GeNN, we performed a series of 10 simulations of 10 s of biological activity of the cortical microcircuit with each simulator, using different seeds for random number generation. The execution time of the simulations can be divided into building time and simulation time of biological activity. The building time includes the time needed to allocate memory for connections, neurons, spike generators, and recording devices, to build connections and to initialize the values of state variables and parameters. Figure 7A shows the building time for NEST and NeuronGPU. On a system equipped with an Intel Core i9-9900 K CPU, the building times were 36.8 ± 0.6 and 39.7 ± 0.4 s for NEST and NeuronGPU, respectively. The building time of NeuronGPU is comparable to that of NEST. This is due to the fact that in NeuronGPU the connections are initially created in the RAM, and only immediately before the simulation they are copied from RAM to GPU memory. The times for code generation, compilation, and initialization of the cortical microcircuit model with GeNN were 49.7, 20.6, and 0.65 s, respectively, as shown in Figure 7B. Importantly, since GeNN uses a code-generation approach, while in NeuronGPU the models are created dynamically, the building times of GeNN and NeuronGPU cannot be directly compared. In GeNN the code of the model is generated from C/C++-like code fragments and it must be compiled before execution. Any changes in the model parameters require a new generation and compilation of the code. Once the code is generated and compiled, the initialization is very fast. Figures 7C,D show the simulation times per unit time of biological activity for NeuronGPU, NEST and GeNN on different CPU and GPU platforms. The simulation time per second of biological time with NEST running on the Intel Core i9-9900K CPU was 62.7 ± 0.3 s. On a system equipped with an NVIDIA Tesla V100 GPU card, the simulation time per second of biological time with GeNN was 2.16 s. NeuronGPU was 31.6% faster than GeNN, with a simulation time of 1.641 ± 0.014 s on the same GPU. On an NVIDIA RTX 2080 Ti GPU card, the simulation time per second of biological time with GeNN was 1.398 ± 0.007 s, while NeuronGPU was 32.5% faster with a simulation time of 1.055 ± 0.004 s. Figure 7E shows the contributions of neuron dynamic update time, Poisson generator time and spike handling and delivery time to the total simulation time of the Potjans-Diesmann model, simulated using NeuronGPU and GeNN on a Tesla V100 GPU. It should be noted that while in the case of NeuronGPU the Poissonian input signal is generated by external Poisson spike generators connected to the neurons, in the case of GeNN this is generated within the same code that manages the neuron's dynamics. Furthermore, in the case of GeNN it was not possible to separate the time used for spike handling and delivery from the remaining contributions to the simulation time. In the case of NeuronGPU, excluding the neuron dynamic update time and the Poisson generator time, most of the remaining simulation time is spent on handling and delivering the spikes. Assuming this is also the case with GeNN, the improvement in the simulation time of NeuronGPU over GeNN would be mainly due to a more efficient approach in spikes handling and delivery.

### 3.2. Simulation of the AdEx-Neurons Balanced Network Model

**Figure 8:**
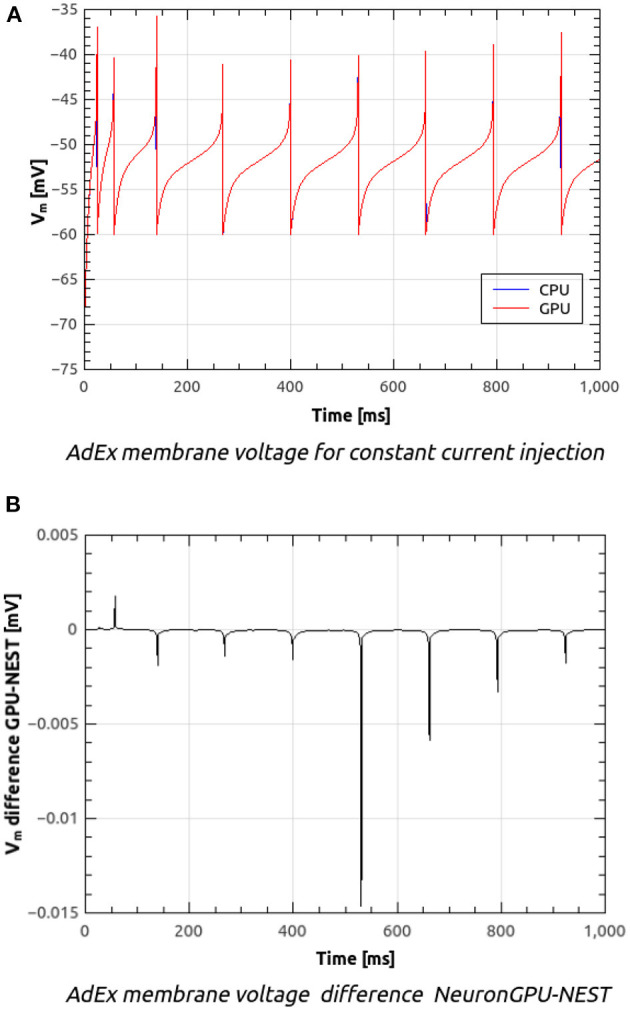
Membrane voltage of an AdEx neuron with the parameter values reported in Table 2 and an injected current of 700 pA, simulated with NeuronGPU and with NEST **(A)** and difference between the two simulation signals **(B)**.

**Figure 9:**
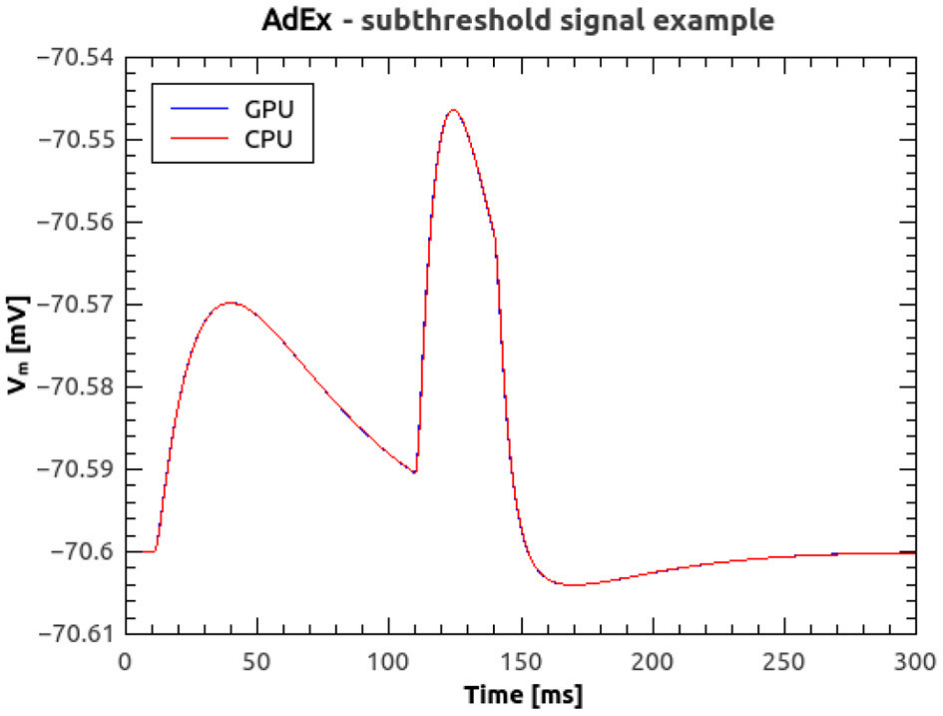
Membrane voltage of an AdEx neuron stimulated by three input spikes in a subthreshold condition, simulated using NeuronGPU and NEST.

Figure 8A shows the time course of the membrane voltage of an AdEx neuron with the parameter values reported in Table 2 and an injected current of 700 pA, simulated with NeuronGPU and with NEST. With the exception of the peaks, the two plots appear to be perfectly superimposed on this scale. Figure 8B represents the difference between the two signals simulated with NEST and with NeuronGPU. Apart from the peaks, the difference is in the order of a few 10^−4^ mV. Figure 9 shows the time course of the membrane voltage of an AdEx neuron stimulated by three input spikes on three different receptor ports in a subthreshold condition, simulated with NeuronGPU and with NEST.

**Figure 10:**
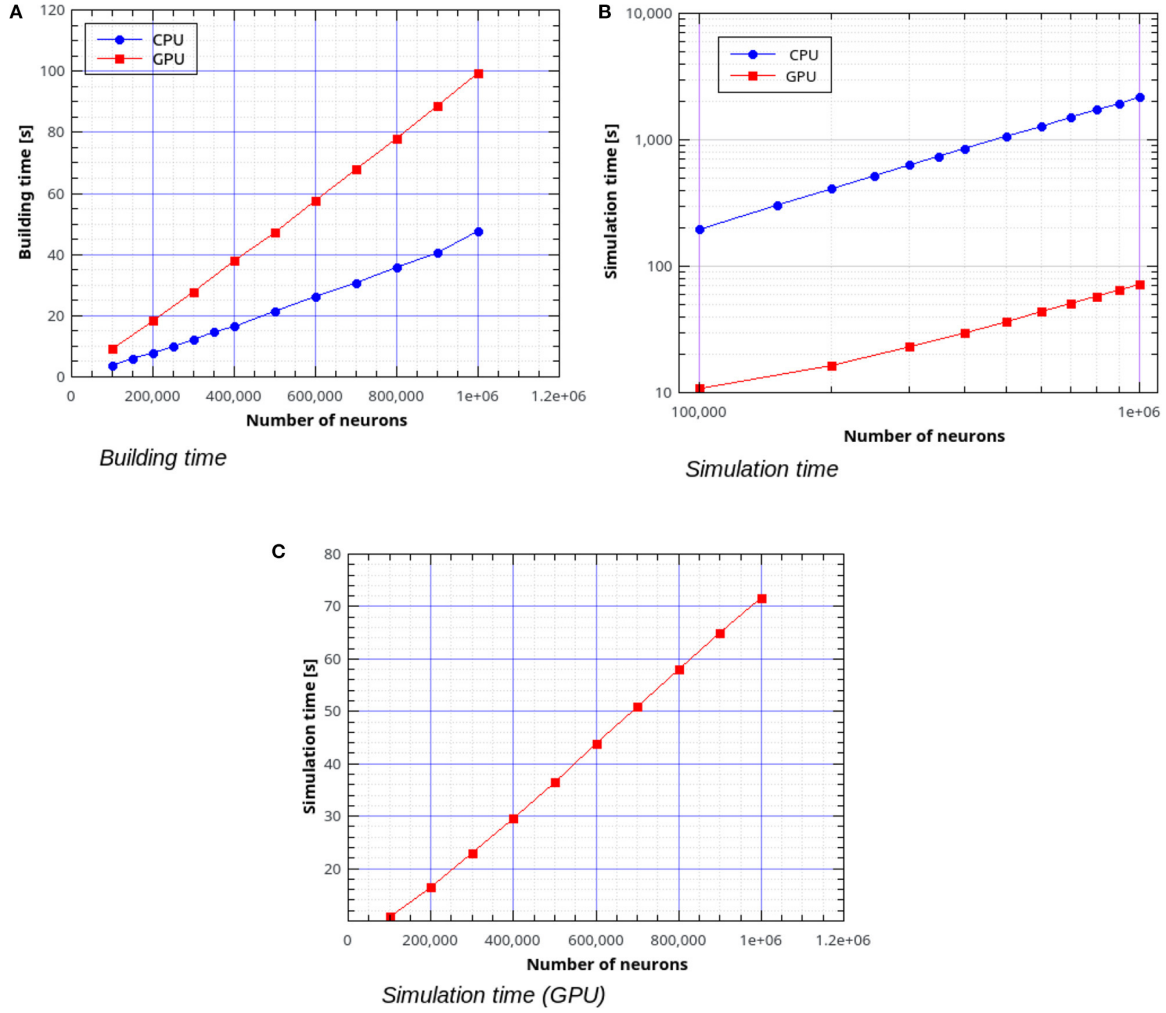
Building time **(A)** and simulation time **(B)** for the balanced network simulations with a variable number of neurons and a fixed number of 1,000 input connections per neuron, simulated using NeuronGPU and NEST, and simulation time for NeuronGPU shown on a different scale **(C)**. The time step for spike communication is set to 0.1 ms.

In the remaining part of this section we present the results of simulations of the AdEx-neurons balanced network with the parameters shown in Tables 1, 2. Figure 10A shows the building time for the balanced network simulations as a function of the number of neurons, for a fixed number of 1,000 input connections per neuron. Figures 10B,C represent the simulation time per second of biological activity of the balanced network as a function of the total number of neurons. It can be observed that the GPU simulations are faster than the CPU's by a factor ranging from about 18× for 100,000 neurons with 10^8^ connections to 30.4× for 10^6^ neurons with 10^9^ connections.

**Figure 11:**
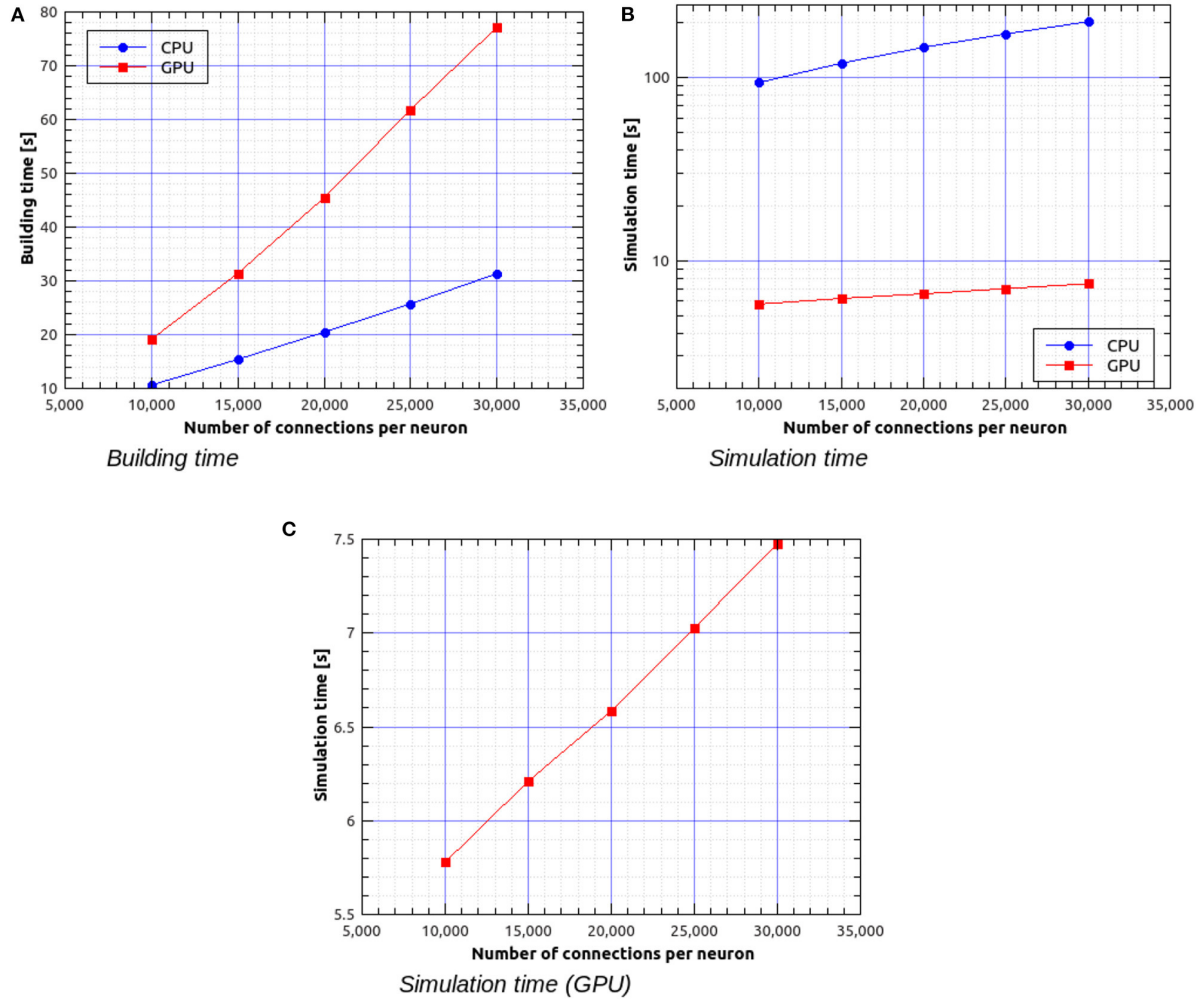
Building time **(A)** and simulation time **(B)** for the balanced network simulations with a fixed number of 30,000 neurons and a variable number of input connections per neuron, simulated using NeuronGPU and NEST, and simulation time for NeuronGPU shown on a different scale **(C)**. The time step for spike communication is set to 0.1 ms.

Figure 11A shows the building time as a function of the number of connections per neuron for a fixed total number of neurons, which was set to 30,000. Figures 11B,C represent the simulation time as a function of the number of connections per neuron. It can be observed that, in this case, simulations on GPU are faster than on CPU by a factor ranging from about 16× for 30,000 neurons with 3 · 10^8^ connections to about 27× for 30,000 neurons with 9 · 10^8^ connections.

### 3.3. Simulation of the Izhikevich-Neurons Balanced Network With STDP Synapses

**Figure 12:**
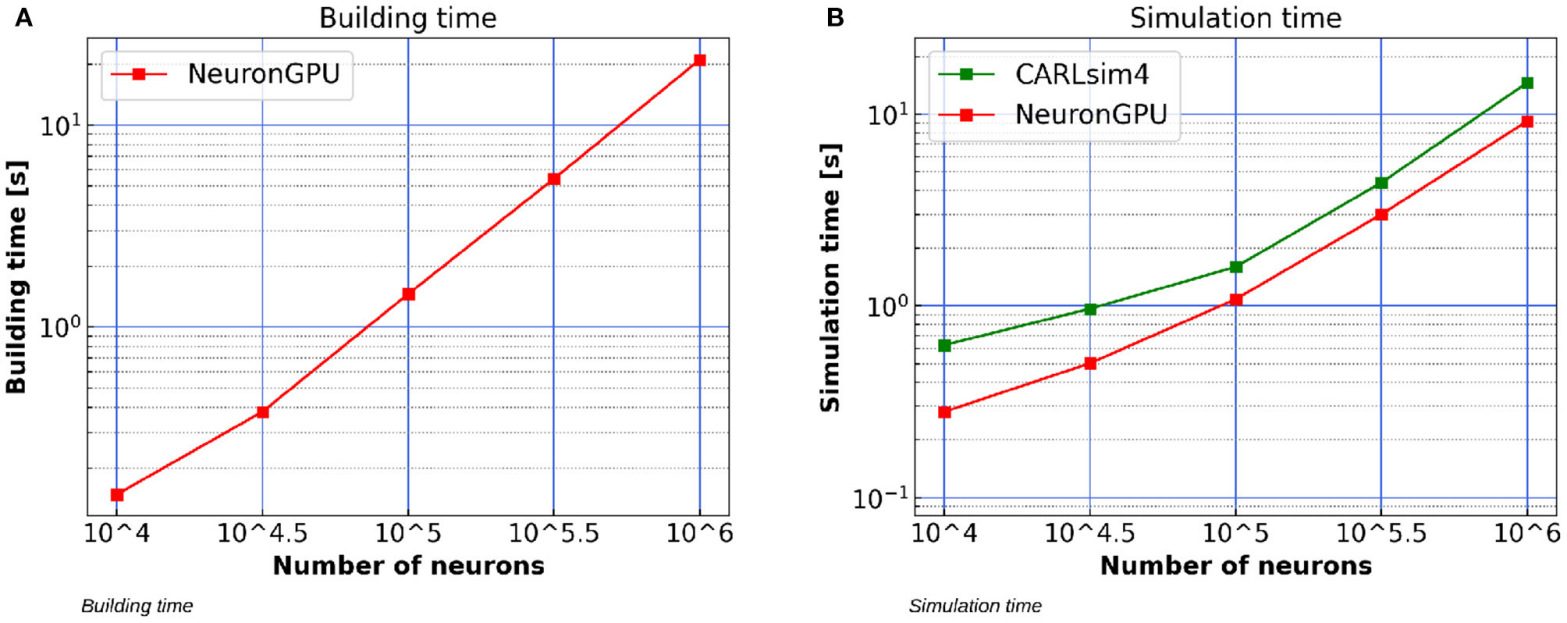
**(A)** Building time of the Izhikevich-neurons balanced network model as a function of the number of neurons, simulated using NeuronGPU on a system equipped with a Tesla K80 GPU. **(B)** Simulation time per second of biological activity of the Izhikevich-neurons balanced network model simulated using NeuronGPU and CARLsim 4 on a Tesla K80 GPU. The data for the latter are taken from Chou et al. (2018). STDP plasticity is active on excitatory connections. The simulation time step is set to 1.0 ms.

Figure 12A shows the building time of the Izhikevich-neurons balanced network model as a function of the number of neurons, simulated using NeuronGPU on a system equipped with an Intel Xeon E5-2686 v4 processor, 64 GB RAM and a Tesla K80 GPU. Figure 12B compares the simulation time per second of biological activity of the model simulated using NeuronGPU with that of CARLsim 4. The simulation times for the latter are taken from Chou et al. (2018), which reports that the simulations were performed on a system that was also equipped with a Tesla K80 GPU card, while the CPU model and the amount of RAM of the system are not specified. It can be observed that the simulation time of NeuronGPU is lower than that of CARLsim 4 in the considered interval. In particular, for 10^6^ neurons and 10^8^ connections NeuronGPU is about 59% faster than CARLsim 4.

## 4. Discussion

As it can be observed in Figure 7, the building time of the cortical microcircuit model simulated using NeuronGPU is comparable to that of NEST, mainly because in NeuronGPU the connections are created in the RAM and only immediately before the simulation loop they are copied to the GPU memory. Compared to most GPU-based simulators, NeuronGPU offers a wide range of choices for connection rules and connection parameter distributions, which can be exploited at runtime and interactively through the Python interface. It is easier to manage these connection rules and these distributions on the CPU side, also thanks to the functions provided by the standard C++ library. In both NEST and NeuronGPU the model parameters, the neuron populations and the network architecture are defined at runtime and the memory they need is allocated dynamically. On the other hand, GeNN uses a code-generation approach. The model parameters, neuron populations and architecture are defined using code fragments similar to C/C++, from which the CUDA/C ++ code of the model is generated. This code must be compiled before execution. Any changes in the parameters, neuron populations or network architecture require a new generation and compilation of the code. Once the code is generated and compiled, the initialization is very fast as it is carried out directly by the GPU with parallel computing algorithms. On the other hand, NeuronGPU achieved a simulation time per second of biological activity of 1.64 s on an NVIDIA Tesla V100 GPU and of 1.055 s on an NVIDIA RTX 2080 Ti GPU, about 32% faster than GeNN, 59x faster than NEST and very close to biological time. Moreover, NeuronGPU was about 59% faster than CARLsim 4 in terms of simulation time per second of biological activity in the simulation of the Izhikevich-neurons balanced network with 10^6^ neurons and 10^8^ STDP synaptic connections. The building time of the AdEx-neurons balanced network simulated using NeuronGPU was about twice as large as that of NEST. However, NeuronGPU was faster than NEST in terms of simulation time per second of biological activity by a factor ranging from about 16× for smaller networks to about 30× for networks with 10^9^ connections. In future releases of the library, the building time could significantly be reduced by creating the connections directly in the GPU memory, exploiting the parallel computing capabilities of the GPU and avoiding the bottleneck of memory transfer from RAM to GPU memory. Besides the relatively long building time, NeuronGPU has other limitations compared to other GPU simulators. In particular, it currently does not include multi-compartment models. The only type of synaptic plasticity available is nearest-neighbor STDP. Neuromodulation is also not included. Multi-GPU simulations are only supported via MPI, which is yet to be evaluated. User defined models are supported, however there is currently no dedicated interface to configure them; the list of state variables and parameters and the differential equations of the dynamics must be modified directly in the code, which has to be recompiled. On the other hand, the high simulation speed demonstrated by the proposed library, significantly higher than that of other CPU and GPU based simulators, combined with the availability of a wide range of neuron models, spike generators, recording tools, and connection rules, makes this library particularly useful for simulations of large spiking neural networks over relatively long biological times. NeuronGPU was recently proposed for being integrated with the NEST neural simulator (Golosio et al., 2020). The high degree of similarity between the Python interfaces of NEST and NeuronGPU immediately simplifies porting scripts from one simulator to the other, and opens the door to integration and cosimulations between NEST and NeuronGPU.

## Data Availability Statement

The datasets presented in this study can be found in online repositories. The names of the repository/repositories and accession number(s) can be found at: https://github.com/golosio/ngpu_cortical_circuits_paper; https://github.com/golosio/NeuronGPU.

## Author Contributions

BG, GT, and PP wrote the manuscript. BG is the main developer of NeuronGPU. BG and PP designed the experiments. All authors contributed to conducting the experiments, analyzing the results, and reviewed the manuscript.

## Conflict of Interest

The authors declare that the research was conducted in the absence of any commercial or financial relationships that could be construed as a potential conflict of interest.
